# General practitioners’ views on quality markers for children in UK primary care: a qualitative study

**DOI:** 10.1186/1471-2296-13-92

**Published:** 2012-09-14

**Authors:** Peter J Gill, Jenny Hislop, David Mant, Anthony Harnden

**Affiliations:** 1 Department of Primary Care Health Sciences, University of Oxford, Radcliffe Observatory Quarter, Woodstock Road, Oxford, OX2 6GG, UK; 2 Faculty of Medicine and Dentistry, University of Alberta, Edmonton, AB, Canada

**Keywords:** Child health, Quality markers, Primary health care, Qualitative research, General practice

## Abstract

**Background:**

Children make up about 20% of the UK population and caring for them is an important part of a general practitioner’s (GP’s) workload. However, the UK Quality Outcomes Framework (pay-for-performance system) largely ignores children – less than 3% of the quality markers relate to them. As no previous research has investigated whether GPs would support or oppose the introduction of child-specific quality markers, we sought their views on this important question.

**Methods:**

Qualitative interview study with 20 GPs from four primary care trusts in Thames Valley, England. Semi-structured interviews explored GPs’ viewpoints on quality markers and childhood conditions that could be developed into markers in general practice. Interviews were audiotaped and transcribed verbatim. Analysis was thematic and used constant comparative method to look for anticipated and emergent themes as the analysis progressed.

**Results:**

All the GPs interviewed supported the development of ‘benchmarks’ or ‘standards’ to measure and improve quality of care for children. However no consensus was expressed about the clinical conditions for which quality markers should be developed. Many participants reflected on their concerns about unmet health care needs and felt there may be opportunities to improve proactive care in ‘at risk’ groups. Some expressed feelings of powerlessness that important child-relevant outcomes such as emergency department visits and emergency admissions were out of their control and more directly related to public health, school and parents/carers. The importance of access was a recurrent theme; access to urgent general practice appointments for children and GP access to specialists when needed.

**Conclusion:**

The GPs expressed support for the development of quality markers for the care of children in UK general practice. However, they flagged up a number of important challenges which need to be addressed if markers are to be developed that are measureable, targeted and within the direct control of primary care. Easy access to primary and secondary care appointments may be an important benchmark for commissioners of care.

## Background

Children are an important part of general practice and comprise approximately 20% of a general practitioners’ (GPs’) patient population [1]. However, less than 3% of the UK’s pay-for-performance markers in the Quality and Outcomes Framework (QOF) focus on them and many of the current markers actively exclude children [2,3]. The lack of markers in primary care reflects in part the difficulties in measuring the quality of care in children [4] and the lack of professional consensus regarding which markers should be implemented in UK general practice [5].

Since being introduced in 2004, the QOF has been successful in raising care quality for specific chronic diseases [6] but there is also evidence that non-incentivised areas of clinical care receive less attention leading to a fall in quality [7]. In particular, studies have highlighted areas where the primary care of children could be improved, such as improving the management of children with chronic conditions [8] and reducing the rising burden of emergency department (ED) visits [9] and unplanned hospital admissions [10]. Several reports have called for the development and implementation of quality markers for children within the QOF [3,8,11].

Earlier studies have explored GPs’ views after the introduction of the QOF including its effect on patient consultations in adults, internal motivation of GPs and unintended consequences in general practice [12-15]. Further, while previous research has addressed the quality of care of children in general practice in relation to specific conditions [16,17] and reported health care professionals’ views on keeping children out of hospital [18], no previous research has investigated whether UK general practitioners would support or oppose the introduction of child-specific quality markers to the QOF. We therefore sought GPs’ views on this important question, including their views on the difficulties likely to be encountered and the aspects of clinical care issues that should be used as markers.

## Methods

### Recruitment

We contacted the Thames Valley Primary Care Agency and requested an updated list of all general practitioners. Using this list and practice knowledge in the department, we used purposeful sampling [19] to identify a maximum variation sample in age, gender, urbanisation, patient populations of different socioeconomic levels and ethnic minority groups of GPs from five Primary Care Trusts (East Berkshire, West Berkshire, Buckinghamshire, Milton Keynes and Oxfordshire) in England. We sent invitation letters to 165 GPs in total, of whom 23 responded. We subsequently interviewed twenty GPs between October 2010 and June 2011 (Table 1), with interviews arranged at a location of choice. Consistent with qualitative methodology, the number of participants was not intended to be numerically representative and the final sample of GPs included practice populations with high levels of deprivation, high percentage ethnic minority and of varying sizes (Table 1).

**Table 1:** Characteristics of the study practices’ patient population and the general practitioners interviewed

**Characteristics**	**(n)**
Study practices’ patient population (n = 20)*	
*Number of general practitioners in practice (FTE)*	
2	2
3–5	9
6–8	6
≥9	3
*Number of nurses in practice (FTE)*	
1–2	8
3–4	7
5–6	5
*Number of patients registered in practice*	
<5,000	5
5,000–9,999	5
10,000–14,999	7
≥15,000	3
*Deprivation*	
<10%	9
10–19%	3
20–29%	0
≥30%	5
Unsure	3
*Ethnic minority*	
<10%	8
10–19%	4
20–29%	3
≥30%	5
***Participants (n = 20)***	
*Sex*	
Female	8
Male	12
*Age, years*	
<40 years	4
40–49 years	8
50–59 years	7
≥60 years	1
Mean (range)	47 (32–62)
*Number of years since qualified*	
<5	3
5–14	5
15–24	5
≥25	7
*Number of patients seen per day*	
<30	4
30–49	11
≥50	5

*Demographic information based on general practitioners’ response.

FTE, full time equivalent.

### Interviews

Following an open-ended question, a semi-structured interview schedule was used to explore key topics in greater depth based on the following domains of enquiry:

▪ Role of practitioners in the care of children

▪ General practitioners’ viewpoint on child health

▪ Perceptions of quality of care provided to children in UK general practice

▪ Use of quality markers in general practice

▪ Quality markers for children in primary care

▪ Implications of quality markers on the care of children

▪ Childhood conditions to focus quality marker development

The interview schedule was developed based on the a priori research questions and informed by current knowledge of quality markers in the UK. The schedule was revised as data collection generated new issues and areas to be explored. The authors include a doctoral student (PG) and non-clinical (JH) and clinical (DM and AH) researchers with an interest in quality of care of children within general practice. The interviews were completed by PG, lasted between 30 and 60 minutes (mean, 46 minutes), were audio-recorded and transcribed fully. After consultation with researchers experienced in qualitative methods (JH), we defined data saturation and the stopping criterion as being when 3 consecutive interviews failed to contribute new themes or ideas [20]. Data saturation was reached after 17 interviews and validated by conducting a further three interviews which produced no additional codes or emergent themes.

### Analysis

Each transcript was read and re-read to gain an overall understanding of the participants’ views and experiences. We used thematic analysis to analyse the interviews. This approach is strongly influenced by Grounded Theory, often being referred to as modified Grounded Theory. The data analysis began after the first interview was transcribed and proceeded simultaneously with the data collection [21]. We used the constant comparison method to interpret the data, looking for anticipated and emergent themes as the analysis progressed [21-26]. We also sought and discussed negative cases [25]. Two authors (PG and JH) independently coded the first two transcripts to produce a coding structure. Subsequently, one author (PG) read through the interview transcripts repeatedly and coded them for analysis while a senior qualitative researcher (JH) checked and revised the coded text. Thematic analysis of the data was facilitated by NVivo 9 computer software. We first identified the initial themes and concepts informed both from the literature as well as inductively by immersion in the data. We then labelled and tagged the data by the concepts and themes identified before lastly sorting the data by themes. To write the results, we completed “OSOP” or “one sheet of paper” analysis, an analysis technique that involves reading through each section of data in turn and noting, on a single sheet of paper, all the different issues that are raised by the coded extracts, along with the relevant respondent identification [27].

To maintain confidentiality, quotations reproduced in this paper have been labelled with the participants’ interview number (e.g. GP4).

### Ethics

The Oxfordshire Research Ethics Committee A (10/H0604/42) reviewed the study and gave it a favourable ethical opinion. We obtained informed consent at the time of the interview. The study adheres to the RATS guidelines on qualitative research [28].

## Results

We report here the key themes identified in the interviews with GPs on the role of quality markers in the care of children in UK general practice, the powerlessness and lack of control GPs perceive relating to quality markers, and their suggestions for specific conditions on which to focus marker development.

### Role of quality markers in children

Most GPs supported the development of quality markers for children in primary care. They viewed markers as “benchmarks” or “standards” that evaluated care, identified outliers and reduced inequalities. As one GP observed, markers function to “systematically document the state of health of the child” (GP7).

"‘…because we’ve got no markers of it, or nobody’s actually looked at it, how would we know? We think we’re doing best practice but we have no idea.’ (GP5)"

"‘I suppose by knowing your rates for a whole hosts of conditions…knowing what good quality care could produce, what was regarded as good quality care by whoever set the benchmark as it were, you could see how well or how badly you were doing.’(GP6)"

Participants stated numerous reasons for developing quality markers: the rising prevalence of health conditions and the associated long-term implications; the fact that children should have received the same high quality care and attention as adults in the QOF; and to formalise and ensure consistency of care across the UK.

"‘It’s nice to think quality things happen, but they don’t always just happen do they? They often need encouragement to happen.’ (GP13)"

However, not all physicians responded positively to indicators and we identified deviant cases. For example, one GP believed that quality measurement referred to “political rhetoric” that led to “homogenised mediocrity” (GP8) rather than excellence. A few participants questioned whether a quality marker framework was required for children or whether it should “just happen out of good practice” (GP3).

GPs suggested various ways to measure quality in children including audits, clinical templates, questionnaires, ED visits, hospital admissions and antibiotic prescription rates. A dichotomy of proposed measurement tools emerged between fairly simple markers that reflected standardised clinical care for all patients (e.g. urine dipstick for all suspected urinary tract infections) compared to others that were complex and would vary with local facilities (e.g. community pathway for management of croup). The importance of communication and good recording in the medical record was reinforced several times as the “health disasters you hear about are all about failures of communication and failures of documentation” (GP4). One GP provided an example of a structured approach to missed appointments:

"‘They are sent three appointments, if then on the third appointment they don’t turn up then it would be flagged up to all of us that actually this person hasn’t, that the immunisations are not up to date, and so for someone to have a conversation with them about it. The nurse, if she gets an opportunity would ring them and if she felt that was appropriate, she’d ring them to see you know, that, it’s how far you take it with them to get an impression of whether they’d made a conscious decision.’ (GP16)"

Other suggested ways to measure quality were condition specific templates for consultations that ensured all aspects of care were provided consistently and were documented in the medical record.

GPs explained how they wanted markers that captured the complexity of child health and measured the most important elements in the care of children. However, many of them relate to the importance of the patient consultation, the unique interaction between the child, parent or carer and clinician, and participants did not want these “soft markers” to become “mechanistic” and lose their meaning.

### Powerlessness and lack of control

There are several frameworks used in the literature to classify quality markers. In the QOF, markers are classified according to the Donabedian framework [29] based on measurable items of care, either as structure (e.g. facilities), process (e.g. prescribing) or outcome (e.g. mortality). Most GPs preferred quality markers that related to outcomes over processes and structures, as “what are important are the outcomes” (GP1) yet they raised numerous concerns about their perceived lack of control over the outcome.

"‘If you look at outcomes for example, neonatal and infant mortality, sudden infant death rates, these are relatively hard outcomes which are measurable. The numbers of children who are hospitalised for a specified condition like asthma for example that is a proxy for measuring the true health status. You look at process outcomes like how many children are taken to care, but those kinds of outcomes are very much influenced by factors other than the child’s health or care status.’ (GP7)"

Outcomes such as childhood mortality were rare in general practice and influenced by a multitude of factors which GPs felt limited their applicability. Several participants described a feeling of powerlessness where poor outcomes occurred. GPs discussed many factors that influenced the health of children such as the nature of the patient illness, the health care system, societal factors and the parents/carers. Most of these factors were felt to be difficult to capture in a quality marker.

"‘Whether somebody is admitted with asthma or sent home, obviously in some ways depends on how bad the asthma is, but it is much more likely to depend on how anxious the parents are and the parents level of coping strategies whether that’s innate or maintained by other people. I don’t think it’s got much to do with the quality of the general practice.’ (GP8)"

"‘But it does rely on good education of populations about where to go first because sometimes it’s just a cultural or a lack of knowledge about how health care systems work. And so sometimes you see, especially you know, people who just may be, we’ve got quite a high immigrant population who just literally, just immigrated and who may not realise about how to register with a GP and therefore their only access to health care is kind of via A & E.’ (GP15)"

For example, Table 2 outlines the various domains of powerlessness that GPs perceived regarding hospital admissions in children. A broad consensus emerged among the GPs that it was unreasonable to set quality markers relating to issues such as ED visits or emergency admissions over which they had very little influence.

**Table 2:** Summary of the sources of powerlessness perceived by GPs as preventing them from influencing childhood hospital admissions

	
**Patient**	**▪**Patient morbidity
	**▪**Health service use behaviour
	**▪**Parents request second opinion
	**▪**Caregivers failure to give medication
	**▪**Chaotic lifestyle of caregiver
	**▪**Parents coping strategy
	**▪**Severity of illness
**Health care system**	**▪**Lack of facilities to observe sick children
	**▪**Lack of senior doctors in emergency department
	**▪**Varying quality of out-of-hours care physicians
	**▪**Inadequate health care facilities
	**▪**Varying admission thresholds at hospital
**Societal**	**▪**Education of population
	**▪**Outbreaks of illnesses
	**▪**Bank holidays / vacation dates
	**▪**Conflicting health messages
	**▪**Deprivation and poverty
	**▪**Cultural factors

Several GPs discussed the complexity of primary care particularly in areas of high social deprivation and with large immigrant populations. Participants stated that if markers are linked to payment yet failed to capture the clinical context and the challenges of general practice there may be unintended consequences. GP15, who practices in an urban centre in an area of high deprivation, explained further:

"‘We normally [have] over a hundred a day calls from people saying it’s an emergency, saying they have to be seen that day. So if I see a child, I have to make a judgement, within, often a five minute consultation, because we run five minute consultations, as to whether they’re safe to go home with no treatment, safe to go with treatment, or whether they need observation. And if they need observation then I have to admit them, because I haven’t got the scope to bring them back later in the day to see because we just haven’t got any staffing or any facilities to do that. If we had more staff, then you could probably do that, and they could be avoidable admissions but if you then use that as a marker, especially linked to payment then the funding for the deprived practices goes down further.’ (GP15)"

One participant cited Julian Tudor-Hart’s Inverse Care Law to describe the potential unintended consequences, the principle that “the availability of good medical care tends to vary inversely with the need for it in the population served” [30]. The possibility of incorporating additional features into quality measurement (e.g. parent’s noncompliance with therapy or clinicians’ poor adherence to practice guidelines) raised the practical issue of how that could be measured without creating time consuming activities. Many GPs were resistant to activities that would increase paperwork or result in data collection without clear reasons.

GPs described the differences between adults and children, primarily related to communication, autonomy, confidentiality and patient consent as further evidence for feeling powerless.

"‘There are major issues of consent in children because clearly legally the consent for underage children is eighteen and certainly for children under the age of, under the age of Gillick competence shall we say, the right to give and withhold consent lies with the parents, but nonetheless that assumes that the parents have the best interests of the child at heart which isn’t always the case.’ (GP7)"

Therefore, “clearly it’s not the children’s fault” (GP15) if children defaulted on appointments or had poor outcomes and quality markers must be able to take these factors into account.

### Conditions to focus marker development

GPs discussed a wide range of conditions when evaluating specific areas to develop quality markers yet no consensus emerged on any single area as the most important. Many physicians discussed the importance of good accessibility, both in terms of children consulting general practice and access to secondary care for GPs when they need to refer children.

"‘…in theory if people have got easy access to their own GPs, they shouldn’t be pitching up at A & E and if the service they’ve got at their own GPs is easy to access and good then they shouldn’t be choosing A & E.’ (GP15)"

"‘…access to appropriately trained professionals, easy access by their parents, but also that those professionals should have access to secondary care without barriers and with excellent communications between us and them.’ (GP1)"

"‘I suppose if children turned up at the A & E department during a weekday with screaming earache. To give an extreme example, I don’t think that happens, but I mean if that did happen it would be indicative of the fact or it might be very suggestive of the fact that they couldn’t get an appointment to be seen in their practice quickly enough, and so the parents might be desperate enough to have to feel, well I’ve got to go to the only other place I can get, you know, I can’t access my primary care team, so I’ll have to go to the hospital.’ (GP6)"

Participants conveyed how important it was for parents and children to access appropriately trained health care professionals but also expressed frustration with the lack of secondary care support they often felt. The importance of access emerged repeatedly when discussing specific conditions in children.

Many GPs indicated that there were certain ‘at risk’ groups that would have benefitted from planned proactive care due to their concern about unmet health care needs (e.g. children with chronic illnesses). To assist in managing this group of patients, participants suggested generic or template markers for their care:

"‘In paediatrics you could have a register of those with significant medical history and you could have an indicator saying, ‘These children should be reviewed annually and these checks undertaken’ like we do with the mental health checks in adults. That would, in those children, be quite easy to identify because we have them in the ‘at risk’ groups. Flu for example, so you know, those would be on particular ‘at risk’ group which we could target more specifically.’ (GP3)"

"‘If we started to produce markers which would bring forward a group that we wouldn’t see otherwise, for example learning disabled children. We would invite them to come to an appointment, and they want to come, and it gives you an opportunity, not when they’re sick, just to review things, so hopefully we will see where [there] may be a problem with coordination of care. That potentially may have advantages. Get to know the family better. Maybe the parents learn to trust the GP, rather than necessarily always relying on the secondary care colleague.’ (GP9)"

The GPs suggested several specific quality markers for children and these can be grouped into the five areas illustrated in Table 3. Many of the suggested quality markers were related to structure and process measures despite the fact that paradoxically many of the GPs had previously expressed a view that quality markers should focus on outcomes.

**Table 3:** Examples of quality markers suggested by GPs for acute illnesses, health promotion and practice structure and communication

	
**Acute illness**	
**▪**	All children seen with an acute illness should have good safety netting and parental advice.
**▪**	Every child that presents with a urinary tract infection has had a dipstick urine test 99% of the time.
**▪**	Develop a pathway in the community for the management of mild croup by general practitioners.
**Adolescent health**	
**▪**	Discuss contraception with each patient after giving post-coital contraception.
**▪**	Every sexual health related consultation in under 18 year olds must include discussions on basic contraception and testing, explored child protection issues and recorded the discussion in the patient record.
**▪**	During all consultations with an adolescent, ensure you have the opportunity to meet with them without their parents present and ensure they are aware they can return without their parents.
**Developmental screening**	
**▪**	Measure the height and weight of children annually and plot it on a growth chart.
**▪**	Formalise health checks, such as have 90% of your three year-olds been seen in a practice.
**▪**	Post-natal education of carers (guardian, mother, father) on nutrition, paediatric life support, etc.
**▪**	Questionnaire at key points to be completed by general practitioner or health visitor whether diet was addressed in a reputable way.
**▪**	Child developmental screening checks by the general practitioner, including physical examination, social evaluation and school performance.
**▪**	Appropriate health promotion with children and young people by discussing diet, healthy eating, exercise, smoking, alcohol, sexual health and teenage pregnancies.
**Obesity**	
**▪**	Develop a register of children with a body mass index (BMI) over a certain number.
**▪**	Have education classes about obesity, giving patients advice, referring them to a dietician or having a dietician assess their home and giving the whole family advice.
**Practice structure and communication**	
**▪**	Annual review of all children who default on an appointment.
**▪**	In children that fail to arrive for immunisations, have general practitioners made and enquired to the parents regarding why?
**▪**	Computer flagged up children that consulted >5 times per year for planned review.

The major health promotion related conditions that emerged as important were developmental screening, obesity and adolescent health. In particular, GPs stated that changes to the role of the health visitor have raised questions regarding the responsibilities in developmental screening. The health visitor acted as a liaison with individuals in the field, such as in school, with social services and with parents. GP11 referred to health visitors as “trusted advisors” or “what society used to have…the wise woman” and felt their role was essential and should be re-affirmed.

Many participants stated that the current training system for GPs should be improved. GP1 felt that if the problem with training continued, there would be a “loss of confidence in the ability of GPs to perform safely and effectively with kids.”

"‘So one concern, I would say, I am probably with the Royal College of Paediatrics and Child Health and I do think that there’s a danger that some trainees coming through may not be getting enough exposure to paediatrics.’ (GP3)"

Another GP trainer felt that trainees appeared to be concerned with completing new-born and six-week checks due to the lack of clinical experience. GP9 highlighted the difference in the exposure between adults and paediatric medicine in training:

"‘Some GPs will come in [to general practice] with no paediatric experience at all, whilst essentially virtually no doctor will come into general practice without ever working with adult populations.’ (GP9)"

Clearly there seems to be a role for developing markers focused on training. In nearly all interviews, child protection was discussed as an important area to develop quality measures which required elements of access, training, communication and structure to function effectively. In addition, antibiotic prescriptions emerged as a potential specific quality marker in children; however some GPs expressed concern over using it as a quality marker saying that it may be acceptable for “academic” general practice but not for most GPs. Unfortunately it was not possible to tease out the issues underlying this interesting viewpoint.

## Discussion

Most of the GPs interviewed supported the development and implementation of quality markers for children in UK general practice. Quality markers were seen as important for assessing the current standard of paediatric primary care and improving its future quality. However, they were concerned that they would be judged on outcomes which they felt powerless to influence. It certainly makes no sense to judge GPs’ clinical performance on the basis of outcomes unlikely to be influenced by their clinical activity. It is also important that any quality indicators adopted enjoy professional support.

There was an expressed preference for quality markers based on outcomes rather than on structure and process. However, when pressed to suggest possible markers they often went on to say paradoxically that most important health outcomes are influenced little by the quality of primary care. Consequently, many of the specific quality measures proposed by the GPs were related to process rather than outcome. This paradox is also reported by researchers in the US who noted that the development of outcome-based quality markers for children is particularly challenging because children’s health is affected by so many issues other than the quality of medical care [4]. A good example was the views expressed about emergency admissions. While many saw this as an important outcome which reflects quality, many participants also commented that such admissions in children are due to numerous factors over which GPs have little control (and the evidence supports this view [10,31]). The balance of opinion expressed was therefore that emergency admissions could not be used as a quality marker for the care of an individual practitioner.

While several quality markers for specific clinical conditions were suggested by participants, no consensus emerged about the most important. Quality markers for a number of the conditions proposed have already been developed by non-UK organisations such as the RAND Corporation and the Agency for Healthcare Research and Quality [32,33]. However, notable gaps remain, particularly for topics such as training. Certain initiatives have already commenced to address this gap including the RCGP First5 initiative [34,35]. The new National Health Service Outcomes Framework for 2012/13 also includes health improvement targets aimed at reducing unplanned hospital admissions for children with select chronic conditions (i.e. asthma, epilepsy and diabetes) and select lower respiratory tract infections [36].

The potential to develop quality standards for access to care was mentioned by a number of participants. Although this has been a major focus of primary care reform in the UK over the past decade (which included the introduction of ‘Advanced Access’ and setting a target that general practice had to provide an appointment within 48 hours) success in addressing access barriers has been modest [37]. Lack of easy access to primary care may partly explain the rise in ED visits for children in England [9]. However, access is not an issue specific to children and development of any quality marker would need to avoid adverse consequences for other age groups. The potential for unforeseen adverse consequences of setting quality markers was raised by a number of participants and has been recognised as a good reason for piloting before national roll-out [37].

A number of participants also raised the potential for quality markers to increase social inequality, in one case citing the Inverse Care Law [30]. While the potential is clear for financial incentives based on quality markers to penalise under-achievement caused more by the social deprivation of the practice population rather than the quality of clinical practice (e.g. teenage pregnancy or smoking rates), recently published evidence based on existing QOF markers shows very little systematic difference in achievement of targets in practices in relation to the population deprivation index [38,39]. However, there is a potential to use quality markers as a mechanism to target social inequality in general and specific groups of children at high-risk of ill-health in particular. Only one of the participants specifically suggested such an approach (setting a quality standard for follow-up children that fail to attend appointments) but it clearly is feasible to develop quality markers for ‘at risk’ children to ensure they have a planned proactive review of their care [8].

A potential criticism of the study is the limitation of the sampling frame to the Oxford health region and the likelihood that the general practitioners volunteering to participate had both a particular interest in child health and an enthusiasm to improve care quality. It is therefore possible that we did not identify some views held only by general practitioners without such enthusiasm (who might have been more strongly opposed to the idea of quality markers for care of children). Moreover, general practitioners are not the only clinicians providing care in a community setting and ideally we would have extended the study to include other health professionals (e.g. health visitors, practice nurses and reception staff) and perhaps parents. Nevertheless we did achieve data saturation and identified a wide range of important issues and opinion which should inform quality marker development.

## Conclusions

There was support amongst the GPs interviewed for the development of quality markers for the care of children in UK general practice. However, they flagged up a number of challenges which need to be addressed if standards are to be developed that are measurable, targeted and within the direct control of primary care. Quality markers are much more likely to be successful in driving quality improvement, and to need less financial incentivisation, if health professionals believe that they are a fair and just indicator of the quality of their care. Easy access to primary and secondary care appointments may be an important benchmark for commissioners of care.

## Competing interests

The author declares that they have no competing interests.

## Authors’ contributions

PG conceived and designed the study, carried out the fieldwork, completed the data analysis, wrote the first draft of the article and edited later drafts. JH contributed to study design, data analysis and critical revisions to the article. AH and DM supervised PG, contributed to study design and critical revisions to drafts of the article. All the authors contributed to drafts of the article, commented and contributed to revised drafts of the paper and read and approved the final draft.

## Pre-publication history

The pre-publication history for this paper can be accessed here:

http://www.biomedcentral.com/1471-2296/13/92/prepub
